# Mutations in mitochondrial DNA causing tubulointerstitial kidney disease

**DOI:** 10.1371/journal.pgen.1006620

**Published:** 2017-03-07

**Authors:** Thomas M. Connor, Simon Hoer, Andrew Mallett, Daniel P. Gale, Aurora Gomez-Duran, Viktor Posse, Robin Antrobus, Pablo Moreno, Marco Sciacovelli, Christian Frezza, Jennifer Duff, Neil S. Sheerin, John A. Sayer, Margaret Ashcroft, Michael S. Wiesener, Gavin Hudson, Claes M. Gustafsson, Patrick F. Chinnery, Patrick H. Maxwell

**Affiliations:** 1 Oxford Kidney Unit, Churchill Hospital, Oxford, United Kingdom; 2 Cambridge Institute for Medical Research, University of Cambridge, United Kingdom; 3 Kidney Health Service, Royal Brisbane and Women’s Hospital, School of Medicine, The University of Queensland, Australia; 4 UCL Centre for Nephrology, Royal Free Hospital, London, United Kingdom; 5 MRC-Mitochondrial Biology Unit, University of Cambridge, United Kingdom; 6 Institute of Biomedicine, University of Gothenburg, Sweden; 7 MRC Cancer Unit, University of Cambridge, United Kingdom; 8 Institute of Genetic Medicine, International Centre for Life, Newcastle University, Newcastle upon Tyne, United Kingdom; 9 Institute of Cellular Medicine, Newcastle University, Newcastle upon Tyne, United Kingdom; 10 Department of Medicine, University of Cambridge, United Kingdom; 11 Department of Nephrology and Hypertension, Friedrich-Alexander University Erlangen-Nürnberg, Erlangen, Germany; 12 School of Clinical Medicine, Cambridge University, Cambridge, United Kingdom; Max Planck Institute for Biology of Ageing, GERMANY

## Abstract

Tubulointerstitial kidney disease is an important cause of progressive renal failure whose aetiology is incompletely understood. We analysed a large pedigree with maternally inherited tubulointerstitial kidney disease and identified a homoplasmic substitution in the control region of the mitochondrial genome (m.547A>T). While mutations in mtDNA coding sequence are a well recognised cause of disease affecting multiple organs, mutations in the control region have never been shown to cause disease. Strikingly, our patients did not have classical features of mitochondrial disease. Patient fibroblasts showed reduced levels of mitochondrial tRNA^Phe^, tRNA^Leu1^ and reduced mitochondrial protein translation and respiration. Mitochondrial transfer demonstrated mitochondrial transmission of the defect and *in vitro* assays showed reduced activity of the heavy strand promoter. We also identified further kindreds with the same phenotype carrying a homoplasmic mutation in mitochondrial tRNA^Phe^ (m.616T>C). Thus mutations in mitochondrial DNA can cause maternally inherited renal disease, likely mediated through reduced function of mitochondrial tRNA^Phe^.

## Author summary

Mitochondria provide the cell’s energy through respiration, using glucose and oxygen to produce ATP. Critical components for this process are encoded by maternally inherited mitochondrial DNA. Mutations in mitochondrial DNA usually affect organs that use energy intensively, notably the brain, eyes and muscles. Here we have discovered a variant in the promoter region of mitochondrial DNA in a large family with kidney disease and normal function in other organs. We show that patient-derived mitochondria do not generate energy normally and have reduced promoter activity, leading to loss of mitochondrial gene expression. We also identify similar mutations that might provide a mechanism for other cases of unexplained inherited kidney disease: two families with reduced cellular respiration carry mutations affecting mitochondrial phenylalanine tRNA, normally required for mitochondrial protein translation. Together, this study provides the first example of a disease-causing mutation in the mitochondrial promoter, and also establishes that the kidney is particularly sensitive to different mutations in mitochondrial DNA. As these can arise from a mutation in the promoter region of mitochondrial DNA or in the tRNA itself, and both reduce the amount of mitochondrial phenylalanine tRNA, this may provide a potential unifying mechanism for mitochondrially inherited kidney disease.

## Introduction

End stage renal disease (ESRD) is common, affecting over 660,000 individuals in the United States in 2013 and is increasing in incidence[1]. Renal replacement therapy is life-saving for these patients, but is associated with increased morbidity and mortality, and high cost. In a proportion of cases (currently less than 1 in 10) a genetic cause is identified on the basis of a clear family history and/or specific clinical features. Genetic factors are also presumed to make a major contribution to the development of ESRD in patients without a clear-cut family history of renal disease. Chronic tubulointerstitial nephritis is a significant, but poorly understood, cause of progressive renal disease characterised by bland urinary sediment and histological evidence of interstitial fibrosis and tubular atrophy. To date, mutations in *UMOD*, *HNF1B*, *REN* and *MUC1* have been shown to cause autosomal dominant tubulointerstitial kidney disease (ADTKD)[2]. While these account for a substantial proportion of inherited tubulointerstitial kidney disease, further mechanisms remain to be identified. Here we show that tubulointerstitial renal disease in a large family without other clinical features is caused by a substitution in mitochondrial DNA. We found a single base substitution in the promoter sequence directing heavy-strand transcription. Homoplasmic variants in the mtDNA control region are not traditionally considered to be pathogenic[3], but we show that this substitution alters transcription, and reduced levels of mitochondrial tRNA^Phe^. This was accompanied by a defect in mitochondrial protein synthesis and a biochemical defect affecting multiple components of the respiratory chain. Examination of additional families with unexplained tubulointerstitial kidney disease led to the identification of two pedigrees with a substitution that directly alters the sequence and concentration of the mitochondrial tRNA^Phe^., suggesting that defects in mitochondrial transcription or translation can cause tubulointerstitial kidney disease.

## Results

**Fig 1:**
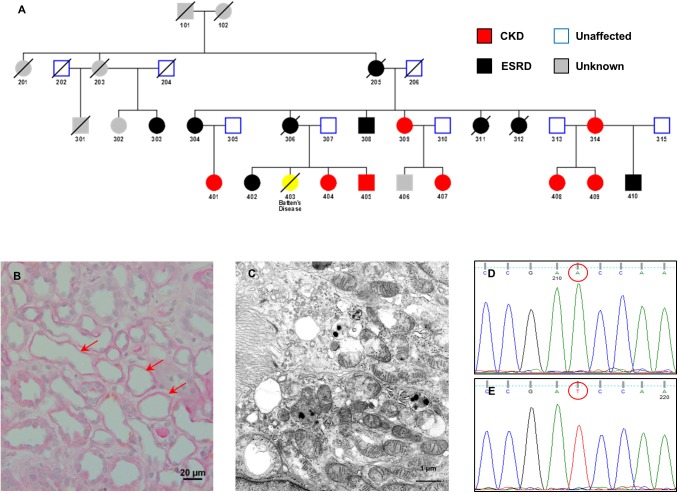
Pedigree with maternally inherited renal disease and m.547A>T substitution. (A) Pedigree of family showing individuals affected with chronic kidney disease (CKD, red symbols), and end-stage renal disease (ESRD, black symbols). One individual (yellow symbol) died many years previously age 18 with a diagnosis of Batten’s disease based on electronmicroscopy of a skin biopsy showing characteristic curvilinear bodies. We consider this to be unrelated to the renal disease (B) Renal biopsy showing evidence of focal tubular atrophy by light microscopy (arrowed). (C) Renal biopsy showing mitochondria in a renal tubular epithelial cell which appear structurally normal on electron microscopy. (D) Sanger sequencing of mtDNA from a control individual. (E) Sequence of an affected individual showing the homoplasmic m.547A>T substitution.

We investigated the inheritance of renal disease in a large multiply affected white English family (Fig 1). Affected individuals developed progressive renal impairment from early adulthood, with increased urinary N-acetyl-beta-D-glucosaminidase (NAG) excretion (NAG to creatinine ratio 73.9–183.8 μmol/h/g, normal < 28), without amino aciduria or renal Fanconi syndrome. Renal biopsy tissue was available from four individuals, showing changes consistent with tubulointerstitial kidney disease. Three individuals had received renal transplants with no evidence of recurrent renal disease up to 26 years following transplantation. Across the pedigree, penetrance of significant renal disease by the fourth decade of life was 100%, although the age of onset and speed of progression varied between individuals. Physical and intellectual development has been normal. Neurological examination in a national referral centre for mitochondrial disease was normal, and imaging of the CNS did not reveal any distinctive features.

Multipoint linkage analysis failed to identify a shared chromosomal haplotype, using both autosomal dominant and X-linked models of inheritance (S1 Fig). This was confirmed by conflicting homozygosity analysis of SNP data, which demonstrated no regions >1.0 cM that were identical by descent in affected individuals, even allowing for a 20% phenocopy rate[4]. Whole exome sequencing of two affected individuals failed to demonstrate any shared substitutions or variants that were predicted to be deleterious in genes associated with ADTKD. No cytosine insertions were identified in the *MUC1* tandem repeat sequences by probe-extension assay.

Given the absence of paternal transmission in the pedigree, we sequenced the mitochondrial genome in urinary epithelial cells from affected patients. Mitochondria contain a 16.5 kb circular genome, encoding two mitochondria-specific ribosomal RNAs, 22 tRNAs and 13 transmembrane proteins which form part of the core of the multi-protein oxidative phosphorylation (OxPhos) complexes I, III, IV and V [5]. Two promoters in the control region drive transcription of the polycistronic light and heavy strand transcripts[6]. We detected a total of 41 benign polymorphisms, one novel insertion located in a small hypervariable region downstream of the light strand promoter (m.16038+1 insC) and a previously unreported substitution (m.547A>T) located within the heavy strand promoter (HSP) of the mtDNA control region (S1 Table)[7]. We focussed our attention on the m.547A>T substitution, which was present at homoplasmic levels in blood, saliva, urinary epithelial cells, and fibroblast samples in all affected individuals [8], in 14 individual skeletal muscle fibres from one patient, and in blood samples taken over 15 years apart. No mtDNA deletions or duplications were detected on long-range PCR of DNA from blood, fibroblast or skeletal muscle.

**Fig 2:**
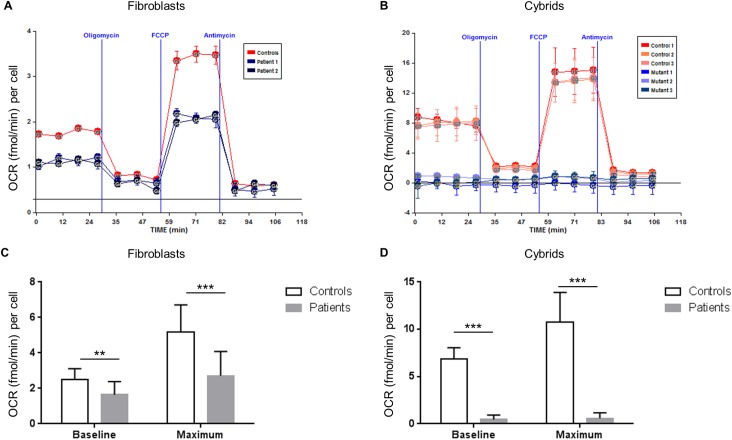
Mitochondrial function is impaired in m.547A>T fibroblasts and cybrids. Oxygen consumption rate (OCR) was measured to assess mitochondrial function in fibroblasts (A, C) and cybrids (B, D), normalised for cell number. Representative comparison of fibroblasts (A) and cybrids (B) from control individuals (red) with fibroblasts or cybrids from patients with the m.547A>T substitution (blue) showing a substantial change in respiratory profile. There was a significant decrease in baseline oxygen consumption, and in maximal oxygen consumption following addition of FCCP in both fibroblasts (C) and cybrids (D). Asterisks *p < 0.05, **p < 0.005, and ***p < 0.001 for control versus patient groups, represented as the mean ± SD of separate experiments performed in triplicate with four patient and four control cell lines.

A diagnostic skeletal muscle biopsy[9] indicated a subclinical reduction in the enzymatic activity of complexes I and IV relative to citrate synthase (S3 Table). Cultured dermal fibroblasts derived from four different patients showed a significantly reduced proliferation rate when galactose was substituted for glucose in the growth medium, suggesting a defect in the mitochondrial electron transfer chain (S2 Fig). Mitochondrial DNA copy number was increased by ~50% (S3 Fig), but citrate synthase activity was not significantly changed relative to control cells (S4 Table). To further characterise the effects of the m.547A>T substitution, we directly measured the oxygen consumption of cultured patient-derived dermal fibroblasts and of trans-mitochondrial cybrids made by fusion of patient platelets (free of nuclear DNA but containing mitochondria) and mitochondria-depleted 143B ρ0 cells[10]. Basal and maximal respiration rates were significantly reduced in fibroblasts and cybrids, confirming a mitochondrially inherited respiratory defect (Fig 2).

**Fig 3:**
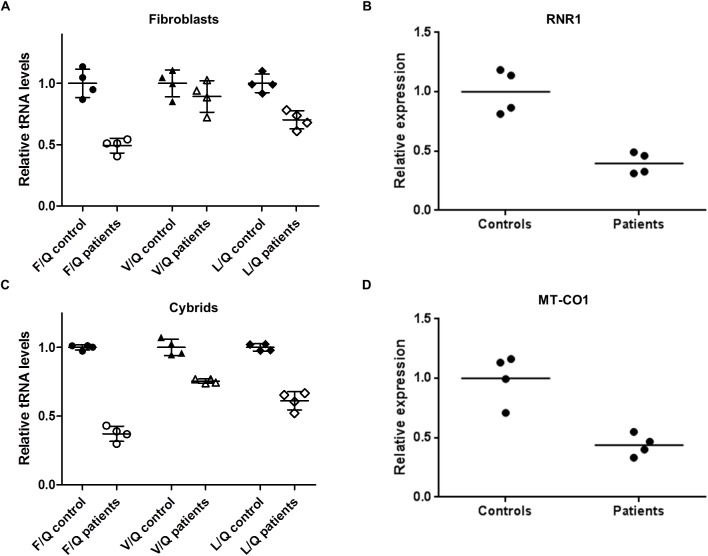
Reduced expression of mitochondrial heavy strand transcripts in m.547A>T patient-derived cells. (A, C) RNA from patient- and control-derived cells (n = 4 each) was analysed for expression of a panel of mitochondrial tRNAs by quantitative Northern blot. The graphs show mean expression of heavy strand tRNAs relative to light strand tRNA Gln(Q). Values are expressed relative to the mean for the control cell lines in each case. Error bars indicate the standard deviation. Heavy strand tRNAPhe and tRNALeu expression is reduced relative to light strand tRNA Gln in m.547A>T fibroblasts (A, p<0.001) and cybrids (C, p<0.001 for tRNA Phe(F), Val (V) and Leu (L)). Experiments were repeated three times with equivalent results. Labelling for 5S rRNA was used to confirm equal loading. Heavy chain transcripts RNR1 (B) and CO1 (D) were measured by quantitative RT-PCR and were reduced in m.547A>T cybrids relative to the light strand transcript ND6. Each point represents the mean of several independent cybrid cell lines derived from a single donor.

To investigate the mechanism of impaired mitochondrial respiration we examined the expression of mitochondrial transcripts in patient-derived and control cells (Fig 3). We observed a significant reduction of heavy strand encoded *RNR1* and *MT-CO1* expression compared to light strand *ND6*, consistent with reduced function of the HSP. We also performed quantitative Northern blot analysis to assess the expression of mitochondrial tRNA species in fibroblasts and cybrids (Fig 3). This demonstrated a highly reproducible reduction in the expression of heavy strand tRNA^Phe^ and tRNA^Leu^ relative to light strand encoded tRNA^Gln^. tRNA^Val^ was also reduced but this was less marked.

**Fig 4:**
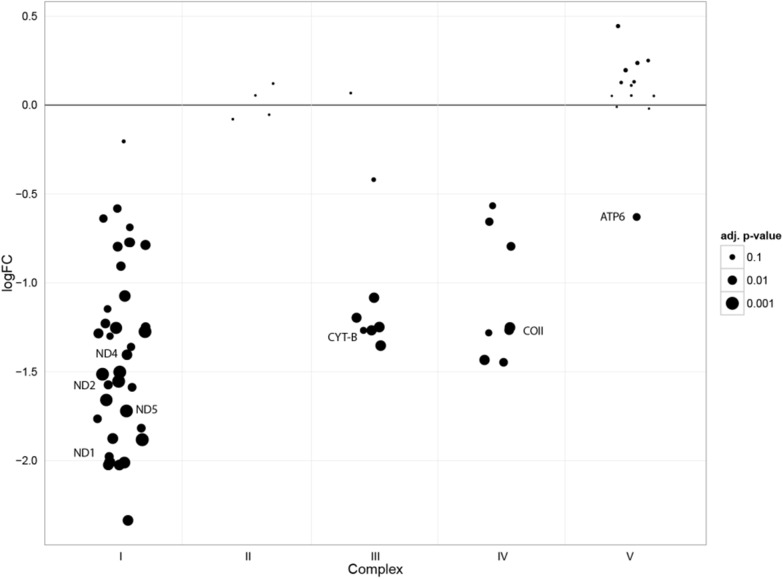
m.547A>T fibroblasts display a marked reduction in complex I, III and IV protein expression. Four individual pairs of fibroblasts from different patients and controls were SILAC labelled and the mitochondrial fraction was analysed by LC-MS/MS. The ratio of protein levels in patient versus control cells is displayed on a log2 scale. Respiratory chain proteins which were quantified in at least two pairs are shown. All identified mitochondrial-encoded proteins are annotated (ND1-5: NADH dehydrogenase subunit 1–5, CYT-B: cytochrome b, COII: cytochrome c oxidase II, ATP6: ATP synthase 6. See S4 Fig for complete annotation). The size of each dot indicates the significance (adjusted p value) of the difference in abundance of the protein in the patient and control samples. Location below the line of equivalence (0.0) indicates lower abundance in the patient samples compared to controls.

To gain insight into effects on mitochondrial protein homeostasis, we developed a quantitative proteomic approach to analyse mitochondrially-enriched fractions of SILAC-labelled fibroblasts, which we term MitoSILAC. This showed a reduction of mtDNA-encoded components in complex I, III, IV, and V in patients (Fig 4, annotated dots), but not in the exclusively nuclear-encoded complex II components. In addition, the majority of nuclear-encoded components of the respiratory chain complexes I, III and IV were also reduced, implying complex instability secondary to a deficiency of mtDNA-encoded subunits. By contrast, nuclear-encoded subunits of complex V (ATP synthase) were unchanged or up-regulated. The reduction of mitochondrially-encoded proteins was also pronounced in patient-derived cybrid lines as assessed by metabolic labelling (S3 Fig).

**Fig 5:**
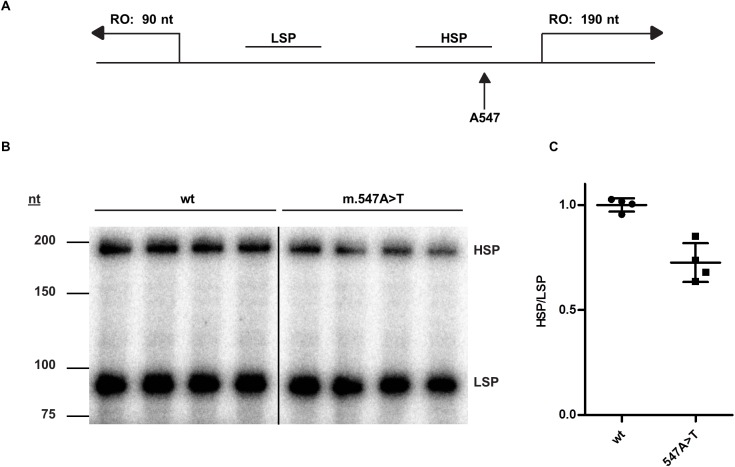
m.547A>T impairs mitochondrial heavy strand promoter activity. (A) In vitro transcription with purified mitochondrial transcription factors and a dual promoter construct containing the heavy (HSP) and light strand promoter (LSP) was used to assess the effect of the m.547A>T substitution. The linear template results in run off (RO) transcripts of 190 nucleotides (nt) from the HSP and 90 nt from the LSP. (B) Metabolically labelled transcripts from wild type (wt) and m.547A>T variant promoter (A547T) were analysed by autoradiography. (C) Analysis of relative band intensities of (B) shows a significant reduction of HSP activity in the presence of the m.547A>T substitution (p = 0.0014). The experiment was performed three times yielding the same result.

To investigate if m.547A>T directly influences HSP activity, we employed a reconstituted *in vitro* transcription system, containing purified mitochondrial transcription factors (Fig 5)[11]. We used a shortened linear mtDNA template that contained both the LSP and HSP. The relative expression of the two different length run-off transcripts allowed direct assessment of HSP transcription relative to the LSP signal (Fig 5A). The m.547A>T substitution decreased HSP transcription activity by 27 ± 7.2% compared to control, confirming a defect in HSP function (Fig 5B and 5C).

**Fig 6:**
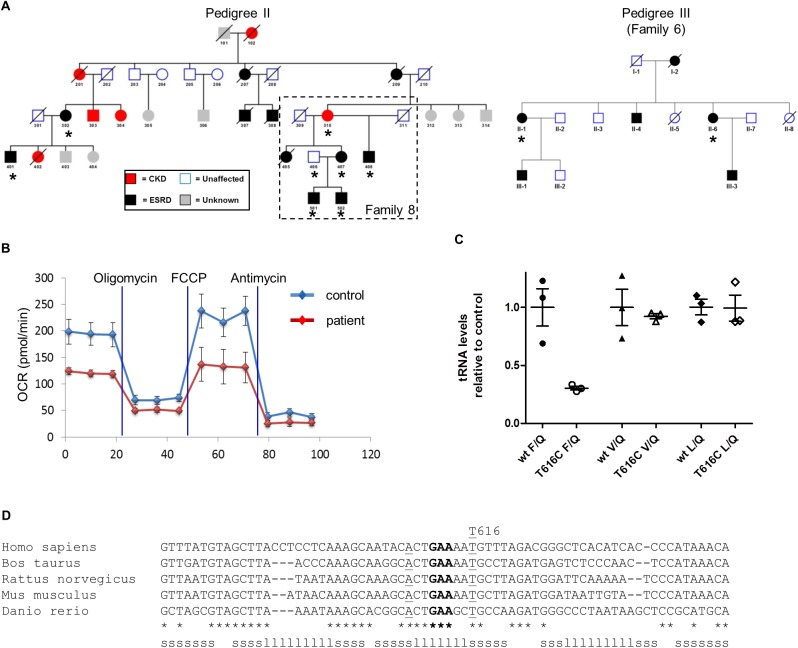
mt.616T>C cybrids have a defect in respiration. (A) Two pedigrees showing potential maternal inheritance of renal disease. Individuals with kidney disease are represented by filled shapes as in Fig 1. Family 8 from ref 13 forms part of pedigree II, and is indicated by the dotted box. Pedigree III is Family 6 from ref 13. Individuals from whom DNA was sequenced in the current study are marked with asterisks. All affected individuals who were sequenced were found to have homoplasmic levels of the m.616T>C substitution. The mitochondrial haplotype is T1a1. (B) Measurement of oxygen consumption in patient-derived and control cybrids showing a reduction in basal (before addition of oligomycin) and maximal respiration (after addition of FCCP) in patient-derived cybrids. (C) Expression levels of tRNAs quantified by Northern blot of three control and patient-derived cybrids show reduced mitochondrial tRNAPhe levels relative to the light strand encoded tRNAGln in mt.616T>C cells.(p = 0.006) The tRNA levels for valine and leucine were unaffected. (D) Conservation of mt tRNA Phe within vertebrates. The anticodon (GAA) is highlighted in bold, the nucleotides forming the last pair of the anticodon stem are underlined. Sequences were aligned with clustal omega and manually adjusted. The stem (s) and loop (l) secondary structure of the tRNA Phe of humans is indicated at the bottom.

Analysis of mitochondrial DNA of 62 other pedigrees with previously unexplained, but potentially inherited, renal disease led to the identification of a different homoplasmic substitution, in the anticodon stem of mt tRNA Phe (m.616T>C) in three families (S2 Table). Two of these families (Pedigrees II and III, Fig 6A) originated from a study of 10 families with tubulointerstitial kidney disease, which only identified a genetic cause in 7 of them[12]. We also found the m.616T>C substitution in a family with tubulointerstitial renal disease which was originally reported in 1982[13]. Further genealogic enquiries demonstrated that this family is actually connected to Family 8 in Ref[12], and the combined kindred is shown in Pedigree II (Fig 6A). Currently we have no genealogic evidence that pedigrees II and III are also connected. However, both are from Australia, and their mtDNA sequence is identical, implying a recent common maternal ancestor. Similar to the family with the m.547A>T HSP substitution, none of the affected individuals in these kindreds had clear evidence of extra-renal disease.

To explore the functional consequences of the tRNA^Phe^ substitution we studied oxygen consumption of patient-derived cybrids which showed reduced basal and maximal respiration, indicating that the patient-derived mitochondrial DNA was responsible for impaired respiration (Fig 6B). Analysis of patient fibroblasts revealed a reduction of the level of tRNA^Phe^ to an extent similar to that which we observed in patients with the HSP substitution (Fig 6C).

## Discussion

We consider that the m.547A>T substitution in the HSP of mtDNA is a pathogenic substitution for the following reasons. First, it segregated with renal disease in 18 members of a maternal lineage and was not seen in 29,867 population controls[7]. Second, it was associated with decreased mitochondrial respiration in skin fibroblasts from multiple affected individuals and in cybrid cell lines. Third, it was associated with deficiency of multiple respiratory chain complexes in a skeletal muscle biopsy from an affected individual and four different fibroblast cell lines, supporting an abnormality of intra-mitochondrial protein synthesis. Fourth, it was associated with reduced *MT-CO1* and mitochondrial tRNA transcripts, consistent with reduced function of the mtDNA HSP. Fifth, it resulted in decreased HSP transcription *in vitro*. Finally, a related mtDNA substitution was identified in other pedigrees with a similar clinical and biochemical phenotype. To the best of our knowledge m.547A>T is the first substitution in the mitochondrial transcriptional control region that has been shown to cause disease. Interestingly, a m.547A>G substitution has been recorded previously without any reported disease association. It is therefore likely that this alternative substitution does not have a functional effect, consistent with the structural impact of such a purine to purine transition being less severe than a purine to pyrimidine transversion [14].

The m.616T>C substitution alters an evolutionarily conserved base, the only pseudouridine in mitochondrial tRNA^Phe^, and will disrupt the last base pairing before the anticodon loop. Reinforcing the pathogenic nature of this substitution, Zsurka et al. described an individual with m.616T>C at near-homoplasmic levels who had intractable epilepsy and developmental delay[15]. Interestingly, she had chronic renal insufficiency when she died at the age of 17, and a maternal relative had died from kidney failure and had epilepsy starting in childhood. Two of the m.616T>C individuals in Pedigree II who are now deceased had epileptic seizures which are described in Burke et al.[13]. Although we have not detected neurological abnormalities in living individuals from this family or in Pedigree III, taken together with the report of Zsurka et al, this suggests that m.616T>C may be associated with a reduced threshold for epileptic seizures. Previous reports of substitutions in mitochondrial tRNA^Phe^ provide some further support for the notion that renal function is sensitive to perturbing this mitochondrial tRNA. Tzen et al. described two siblings with m.608A>G substitutions (the partner nucleotide of 616T in the anticodon stem), neurological features and a urinary concentrating defect[16]. D’Aco et al. reported a child with a heteroplasmic m.586G>A substitution, persistent lactic acidosis, failure to thrive and progressive renal failure[17]. Mitochondrial tRNA^Phe^ is one of two tRNAs that can form a structural component of the mitoribosome[18] which could exacerbate the translational defect. Although minor abnormalities in proximal tubular function are frequent in patients with other mitochondrial mutations, clinically overt renal disease is not usually observed[19]. Taken together with our study, these observations suggest that renal function is particularly susceptible to specific alterations in tRNA^Phe^.

In all the individuals described in this report it is striking that despite the mitochondrial defect being homoplasmic and present in all tissues clinical disease was restricted to the kidney, although we acknowledge that other organs may be affected at a subclinical level. This situation is similar to the organ specific effect of mutations in mt tRNA^Ile^, which primarily cause hypertrophic cardiomyopathy [20][21] and of mutations in NADH dehydrogenase causing Leber’s hereditary optic neuropathy [22]. Taken together, the contrasting effects of these mutations should offer a powerful route to understanding how mitochondrial defects cause disease in different tissues.

From a clinical perspective, we propose the term Mitochondrially Inherited Tubulointerstitial Kidney Disease (MITKD) to complement the recently defined entity, Autosomal Dominant Tubulointerstitial Kidney Disease (ADTKD)[2]. MITKD may account for a significant proportion of unexplained familial tubulointerstitial kidney disease and we recommend that kindreds with compatible patterns of transmission should be offered analysis of mitochondrial DNA as a route to establishing a diagnosis and appropriate genetic counselling.

## Materials and methods

### Genetic analysis

DNA was extracted from peripheral-blood leukocytes, urinary epithelial cells, and saliva. 1 μg of genomic DNA at 75 ng/μl was used for SNP genotyping. Individuals were genotyped using 300,000 single nucleotide polymorphism (SNP) markers with an average separation of 6.1 kb on the Illumina CytoSNP 12 chip (Illumina, Cambridge, UK). Genotyping was performed at the automated, high-throughput system available at UCL Genomics. Linkage analysis was performed on pedigrees using the easyLINKAGE Plus v5.05 graphical user interface for two-/multi-point linkage analysis [23], with analysis by GeneHunter[24] and SimWalk [25]. Pedigrees were confirmed using HaploPainter V.1.043[26] and PedCheck[27]. For calculation of parametric LOD scores a ‘rare dominant’ model was utilised, with disease allele frequency set to 10–4 and disease penetrance vector set to 1.0. Conflicting homozygosity was assessed in this genotype data, using a program written by Dr Daniel Gale and Dr Adam Levine at UCL. mtDNA was analysed by Sanger sequencing. Long-range PCR was performed using published primers[28]. Homoplasmy analysis was performed using labelled amplicons from the D4 primer pair[28] and tetra-primer amplification refractory mutation system PCR designed to detect the m.547A>T substitution.

### Muscle biopsy analysis

A quadriceps muscle biopsy was taken from one affected individual, snap frozen, and analysed using enzyme histochemistry and respiratory chain complex analysis as described[29]. Individual skeletal muscle fibres were isolated by laser capture for quantitative pyrosequencing to determine whether there was any intracellular heteroplasmy[30].

### Generation of cell lines

Fibroblasts from four different patients and four healthy volunteers were obtained by skin biopsy and cultured in Dulbecco’s Modified Eagle’s medium, supplemented with pyruvate and uridine. Cybrids were created by fusion of 143B ρ0 cells and enucleated patient fibroblasts or platelets as described[10]. Cell lines were confirmed to contain the promoter variant by sequencing. All lines tested negative for mycoplasma. All donors consented to research use of their skin-derived primary cells. Volunteers were enrolled at UCL London, study reference 06/Q0406/151.

### Respiration assays

Oxygen consumption rate was determined with a Seahorse XF24 Analyser (Seahorse Bioscience, MA) and normalised for cell number. Respiratory profiles were generated by serial treatment with optimised concentrations of oligomycin (0.5–5 μg/ml), p-[trifluoromethoxy]-phenyl-hydrazone (FCCP) (1–7.5 μg/ml), and antimycin[31] (2–4 μg/ml), with fibroblasts receiving the higher and cybrids the lower concentration. Cell number normalisation was done by sulforhodamine B staining of TCA fixed cells in the seahorse plate as described[32].

### Investigation of mitochondrial gene expression

Quantitative reverse-transcriptase-polymerase chain reaction (qRT-PCR) TaqMan assays (Applied Biosystems, CA) were used to measure the expression of mitochondrially encoded messenger RNA. The following primers were used:

β-Actin forward CCCAGAGCAAGAGAGG, reverse GTCCAGACGCAGGATG;
E-cadherin forward GACAACAAGCCCGAATT, reverse GGAAACTCTCTCGGTCCA;
B2M (RNA) forward GTGCTCGCGCTACTCTCTCT, reverse TCAATGTCGGATGGATGAAA;
MT-CO1 forward ACGTTGTAGCCCACTTCCAC, reverse CATCGGGGTAGTCCGAGTAA;
RNR1 forward CTAGCCACACCCCCACGGGA, reverse CGCGGTGGCTGGCACGAAAT;
ND6 forward AGGTAGGATTGGTGCTGTGG, reverse CCAATAGGATCCTCCCGAAT.

### High resolution Northern blotting

RNA was prepared in Trizol (Invitrogen) according to the manufacturers recommendations, resuspended in formamide and 1 μg separated on a 15% polyacrylamide/TBE gel. RNA was then transferred by wet blotting onto Immobilon NY+ nitrocellulose membranes (Millipore, Watford, UK) and crosslinked in a Stratalinker at 120 mJoules. The crosslinked membranes were transferred to hybridisation tubes and pre-hybridised for an hour at 42°C in hybridisation solution (50% formamide, 10% dextran sulfate, 1% SDS, 5x Denhardt's solution in 5x SSPE buffer). Random-labelled probes were generated by incubating 100 ng PCR amplified tRNA and 5S rRNA templates with 1.85 MBq α-32P dCTP (Perkin Elmer, Seer Green, UK), random decanucleotides and Klenow polymerase exo- fragment (Thermo Scientific DecaLabel DNA Labelling Kit, Waltham, MA). The following primers were used to generate the PCR products:

Phe: 71 nt, Fwd: GTTTATGTAGCTTACCTCCTCA, Rev: GATGTGAGCCCGTCTAAACA
Gln: 69 nt, Rev: AGGACTATGAGAATCGAACCC, Fwd: GATGGGGTGTGATAGGTGGC
Leu 1 (UUR): 75 nt, Fwd: TTAAGATGGCAGAGCCCG, Rev: TTGTTAAGAAGAGGAATTGAACC
Val: 69 nt, Fwd: AGCTTAACACAAAGCACCCA, Rev: TCAGAGCGGTCAAGTTAAG
5S rRNA (RNA5S1): 121 nt, Fwd: GTCTACGGCCATACCACCCTG, Rev: AAAGCCTACAGCACCCGGTAT

Probes were purified on NICK columns (GE healthcare, Sale, UK) and a specific activity of 2x10^6 cpm was added to the membranes in hybridisation solution and incubated over night at 42° C. Up to 3 probes were used together (two tRNAs and 5S rRNA). The membranes were rinsed three times with 2x SSPE at room temperature and once with 2X SSPE/2%SDS for 15 minutes at 65°. Bound probe was quantified by phosphorimaging.

### Mitochondrial DNA promoter assay

The transcription reactions were performed as described previously (Posse et al. 2014) with 100 mM NaCl, 400 nM TFAM, 20 nM POLRMT, 60 nM TFB2M and 4 nM template. The templates used were obtained by restriction cleavage (MscI and HindIII) of pUC18 plasmids containing a mitochondrial DNA insert (human mtDNA 1–741, 547A or 547A>T) generating run-off products of 90 and 190 nucleotides from the light and heavy strand promoters. The α-32P-UTP labelled transcription products were separated on a 4% Urea PAGE and exposed on a phosphorimager plate. HSP transcript levels were quantified and normalized to LSP transcription from the same reaction.

### MitoSILAC

Patient derived fibroblasts were incubated with normal (light) amino acids while control fibroblasts of the same low passage number were incubated with 13C6 Arginine and 13C8 Lysine (heavy) for at least 5 passages. Pairs of patient and control fibroblasts were mixed at 1x10^7 cells each and lysed by dounce homogenisation. Mitochondria were enriched as previously described by differential centrifugation followed by further enrichment on a Percoll gradient[33].

50 μg of enriched mitochondria were resolved approximately 6 cm into a pre-cast 4–12% Bis-Tris polyacrylamide gel (Novex, Thermo Fisher Scientific, East Grinstead, UK). The lane was excised and cut in 8 approximately equal chunks and the proteins reduced, alkylated and digested in-gel. The resulting tryptic peptides were analysed by LC-MSMS using a Q Exactive coupled to an RSLCnano3000 (Thermo Fisher Scientific). Raw files were processed using MaxQuant 1.5.2.8 using Andromeda to search a human Uniprot database (downloaded 03/12/14). Acetyl (protein N-terminus), oxidation (M) and deamidation (N/Q) were set as variable modifications and carbamidomethyl (C) as a fixed modification. SILAC data was loaded in R to process it with the Microarray-oriented limma package to call for differential expression[34], relying on the original normalisation processes produced by MaxQuant as reported previously[35, 36]. The proteomics data was deposited to the ProteomeXchange Consortium via the PRIDE[37] partner repository with the dataset identifier PXD004809. A linear model was fitted with limma for the log-2 normalised expression ratios; moderated t-statistics, moderated F-statistics, log-odds and associated p-values for differential expression were produced with the same package. P-values were corrected for multiple hypothesis testing. Proteins were matched to the different mitochondrial respiratory complexes based on the gene family annotation available at HGNC[38] and plotted using ggplot2 [39]. Low variance of the data between individual donors is indicated by the low incidence of standard deviation greater than 20% as shown in S3 Fig.

### Mitochondrial translation assay

Quantitation of mitochondrial protein translation was done as described by Chomyn[40]. Cells were grown to 80% confluence in 6 well dishes, starved for 20 min in medium without methionine and cysteine, cytosolic translation was blocked with emetine (100 ug/ml) for 30 min before adding 6.8 MBq radiolabelled 35S methionine/cysteine (EasyTag Express35S, Perkin Elmer, Seer Green, UK) for 30 min. Washed cells were harvested and separated on TGX Stain-Free precast gels (Bio-Rad, Marnes La Coquette, France), equal loading was confirmed by UV imaging (ChemiDoc, Bio-Rad) prior drying and exposure to phosphorimager screens

### Statistical analysis

Unless otherwise stated, Student’s t-test was used to assign significance, assuming normal distribution of variance (two-tailed, non-paired). Mean and standard deviation are indicated where appropriate.

### Accession numbers

The traces of the whole mitochondrial sequencing for the m.547A>T variant can be found in the NCBI trace archive, accession number TI 2344039844–2344039995. The mass spectrometry proteomics data have been deposited to the ProteomeXchange Consortium via the PRIDE[37] partner repository with the dataset identifier PXD004809.

## Supporting information

S1 Fig Linkage analysis demonstrates lack of autosomal inheritance. (A) Linkage analysis was performed on all affected individuals (n = 16) with elevated creatinine, using SNP data from the Illumina CytoSNP 12 chip (Illumina, USA). Rare dominant and X-linked models were used and the maximal LOD score (2.64) was considered non-significant given the size of the pedigree (16 individuals which would require a LOD score of 5.1 or above).(B) Linkage analysis on only the ESRD patients (n = 9) also yielded no significant hits (expected LOD score of 3 or above).
(DOCX)

S2 Fig Reduced growth of patient fibroblasts in galactose. Fibroblast lines from four different patients and four healthy controls were cultured with either glucose (filled symbols) or galactose (open symbols) as a carbon source and confluency was measured with an Incucyte HD live cell imaging system. Patient-derived cells in galactose needed a mean value of 40 hours longer to reach 50% confluency compared to glucose. Control cells in galactose reached 50% confluency significantly faster, mean value of 19 hours later than cells supplied with glucose (p = 0.019)
(DOCX)

S3 Fig Increased mtDNA copy number in patient fibroblasts. Mitochondrial copy number was assessed by quantitative RT-PCR for the mitochondrial gene ND1 and the nuclear gene B2M and normalized to the mean ratio of healthy controls. As a group, patient-derived fibroblasts showed a significant increase in copy number relative to control fibroblasts (of different haplotypes).
(DOCX)

S4 Fig m.547A>T cybrids display reduced protein translation. Mitochondrial protein translation was analysed by blocking cytosolic translation with emetine in the presence of ^35^S methionine and cysteine. (A) A clear reduction of mitochondrial protein synthesis was observed in the patient-derived cybrids with the m.547A>T substitution. The characteristic bands of mitochondrial encoded proteins are annotated (ND1-6: NADH dehydrogenase subunit 1–6, CO I-III: mitochondrially encoded cytochrome c oxidase I-III, ATP6: mitochondrially encoded ATP synthase 6). (B) Total protein concentration of the radiolabelled samples was determined using TGX stain free gels (Bio Rad). (C)The scatter plot shows the mean, normalised mitochondrial protein production of patient and control cybrids with error bars indicating the standard deviation (p<0.01).
(DOCX)

S5 Fig Fully annotated mitoSILAC data. Individual subunits of the respiratory complexes identified in at least two of the four mitoSILAC experiments are listed. The binary logarithm of the fold change is shown (LogFC). Error bars are shown where the standard deviation exceeded 20% of the mean. The adjusted p value indicating significant changes is indicated by a blue to red colour scale. Uniprot gene names are given.
(DOCX)

S1 Table mtDNA variants in pedigree I. Base indicates the position relative to the revised Cambridge reference sequence of human mitochondrial DNA; the GB frequency data is derived from 29,867 GenBank sequences with size greater than 15.4kbp and published on the MITOMAP database. A value of 1 indicates 100% prevalence.[7] The mitochondrial haplotype is N1a1a1a, based on 22 indicative mtDNA variants.
(DOCX)

S2 Table mtDNA variants in pedigrees II and III. Base indicates the position relative to the revised Cambridge reference sequence of human mitochondrial DNA; the GB frequency data is derived from 29,867 GenBank sequences with size greater than 15.4kbp and published on the MITOMAP database. A value of 1 indicates 100% prevalence [7].
(DOCX)

S3 Table Muscle biopsy shows reduced complex I and IV activity. Biochemical analysis of skeletal muscle homogenate from a patient carrying the m.547A>T mutation showed that the activities of complexes I and IV are both outside the control range, while complexes II and III activities are normal. All enzyme activities are normalised for citrate synthase activity. Values outside the normal range are shown in bold.
(DOCX)

S4 Table Citrate synthase activity of patient and control fibroblasts. Citrate synthase activity in total cell lysates was assessed by measuring the conversion of oxaloacetate and acetyl CoA to citrate and CoA-SH, which then reacts with dithio-nitrobenzoic acid (DTNB) to yield thio-nitrobenzoate which absorbs at 412 nm[41]. We analysed four different patient and control cell lines (3 technical repeats per experiment and the assay was repeated twice). The ratio of citrate synthase activity in patient and control fibroblast lines did not show a significant difference in any of the assays.
(DOCX)
