# Supplementary material for: Mutations in mitochondrial DNA causing tubulointerstitial kidney disease
Source: PLoS Genet. 2017 Mar 7;13(3):e1006620. doi: 10.1371/journal.pgen.1006620 (PMC5360345; doi:10.1371/journal.pgen.1006620)
Supplement: S1 Fig — (A) Linkage analysis was performed on all affected individuals (n = 16) with elevated creatinine, using SNP data from the Illumina CytoSNP 12 chip (Illumina, USA). Rare dominant and X-linked models were used and the maximal LOD score (2.64) was considered non-significant given the size of the pedigree (16 individuals which would require a LOD score of 5.1 or above).(B) Linkage analysis on only the ESRD patients (n = 9) also yielded no significant hits (expected LOD score of 3 or above). (DOCX) [file pgen.1006620.s001.docx]

**S1A**


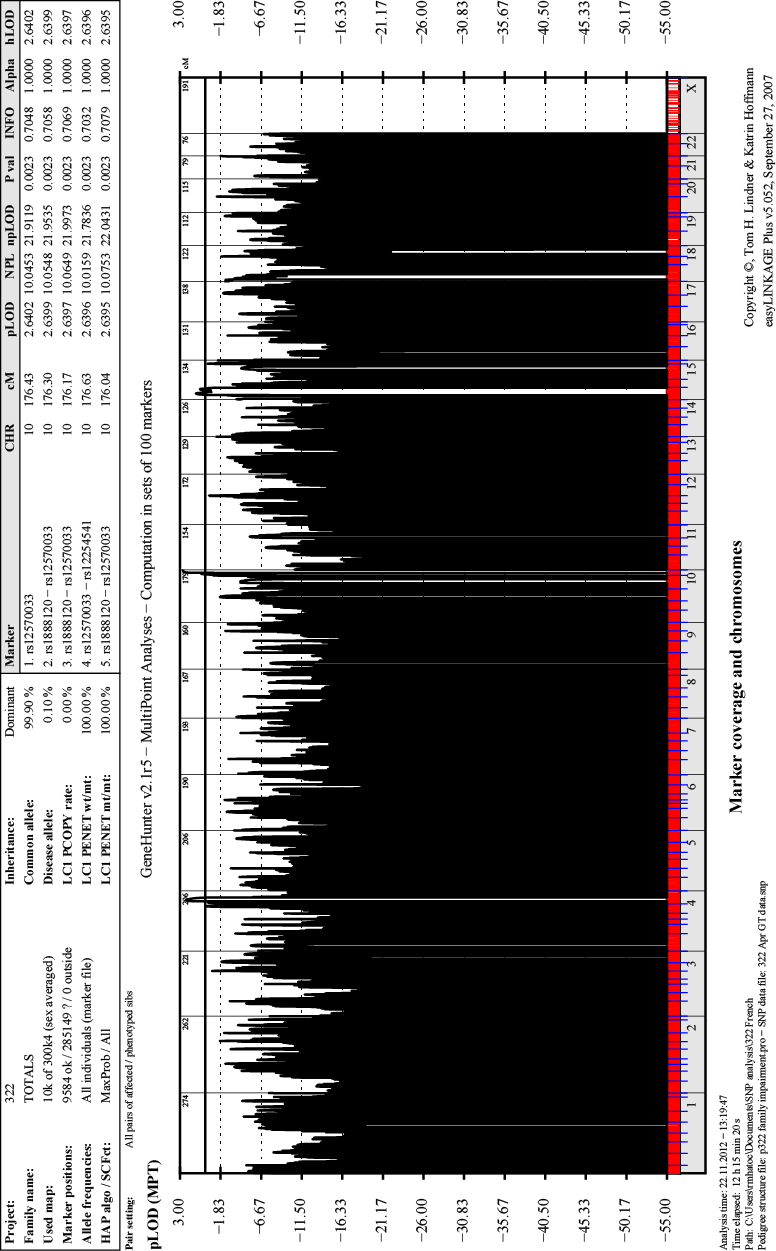


**S1B**


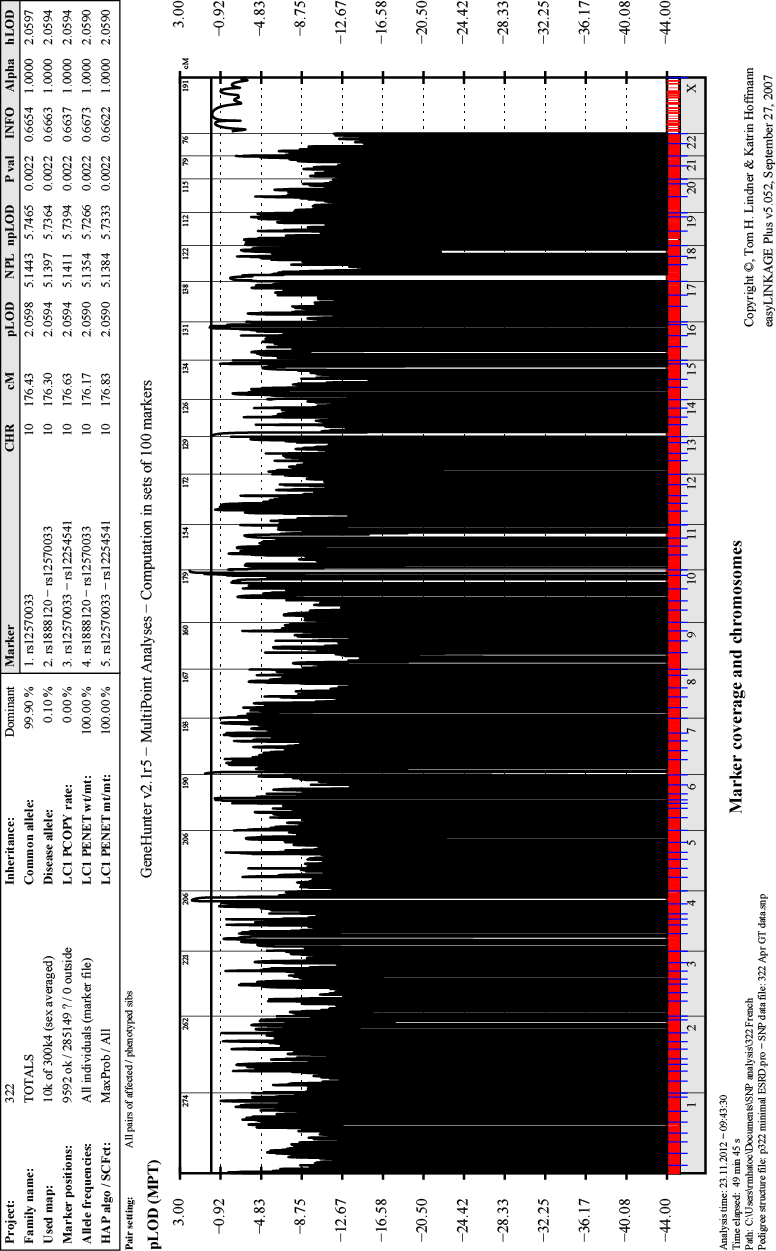


**S1 Figure: Linkage analysis demonstrates lack of autosomal inheritance**

(A) Linkage analysis was performed on all affected individuals (n=16) with elevated creatinine, using SNP data from the Illumina CytoSNP 12 chip (Illumina, USA). Rare dominant and X-linked models were used and the maximal LOD score (2.64) was considered non-significant given the size of the pedigree (16 individuals which would require a LOD score of 5.1 or above).(B) Linkage analysis on only the ESRD patients (n=9) also yielded no significant hits (expected LOD score of 3 or above).
