# Supplementary material for: Mutations in mitochondrial DNA causing tubulointerstitial kidney disease
Source: PLoS Genet. 2017 Mar 7;13(3):e1006620. doi: 10.1371/journal.pgen.1006620 (PMC5360345; doi:10.1371/journal.pgen.1006620)
Supplement: S2 Fig — Fibroblast lines from four different patients and four healthy controls were cultured with either glucose (filled symbols) or galactose (open symbols) as a carbon source and confluency was measured with an Incucyte HD live cell imaging system. Patient-derived cells in galactose needed a mean value of 40 hours longer to reach 50% confluency compared to glucose. Control cells in galactose reached 50% confluency significantly faster, mean value of 19 hours later than cells supplied with glucose (p = 0.019) (DOCX) [file pgen.1006620.s002.docx]

S2 Figure

A


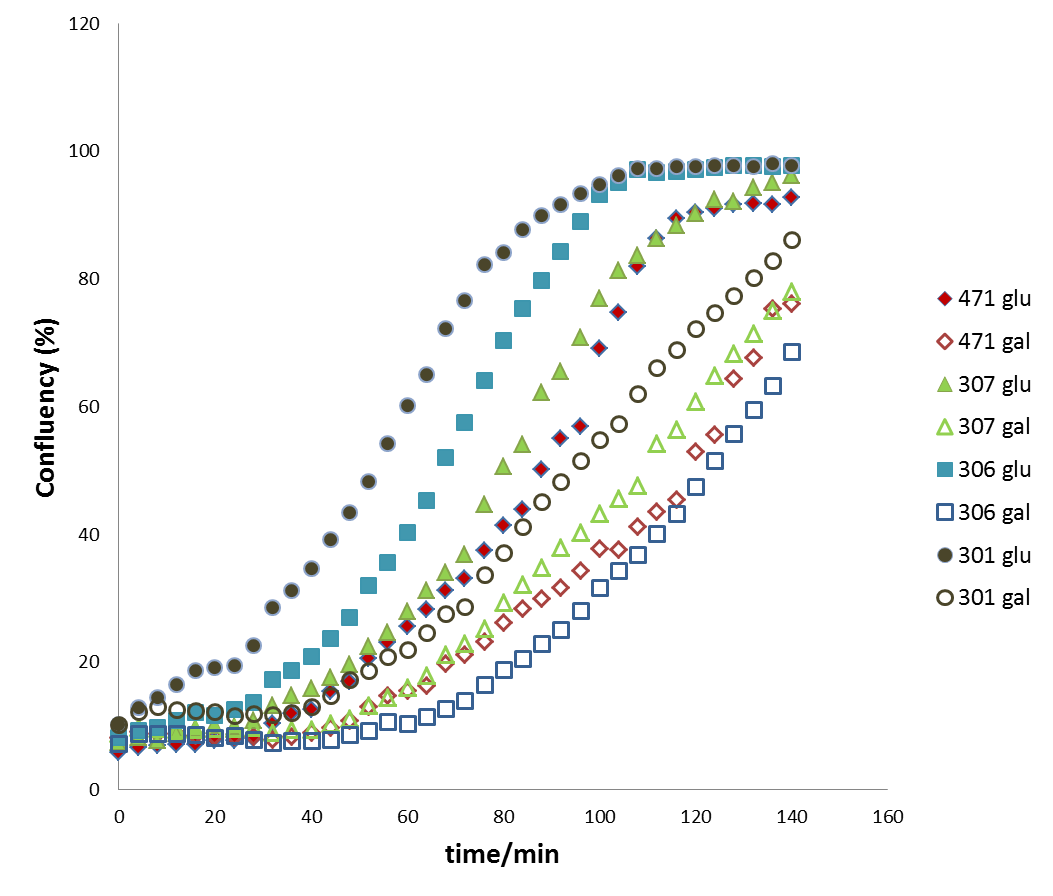


B


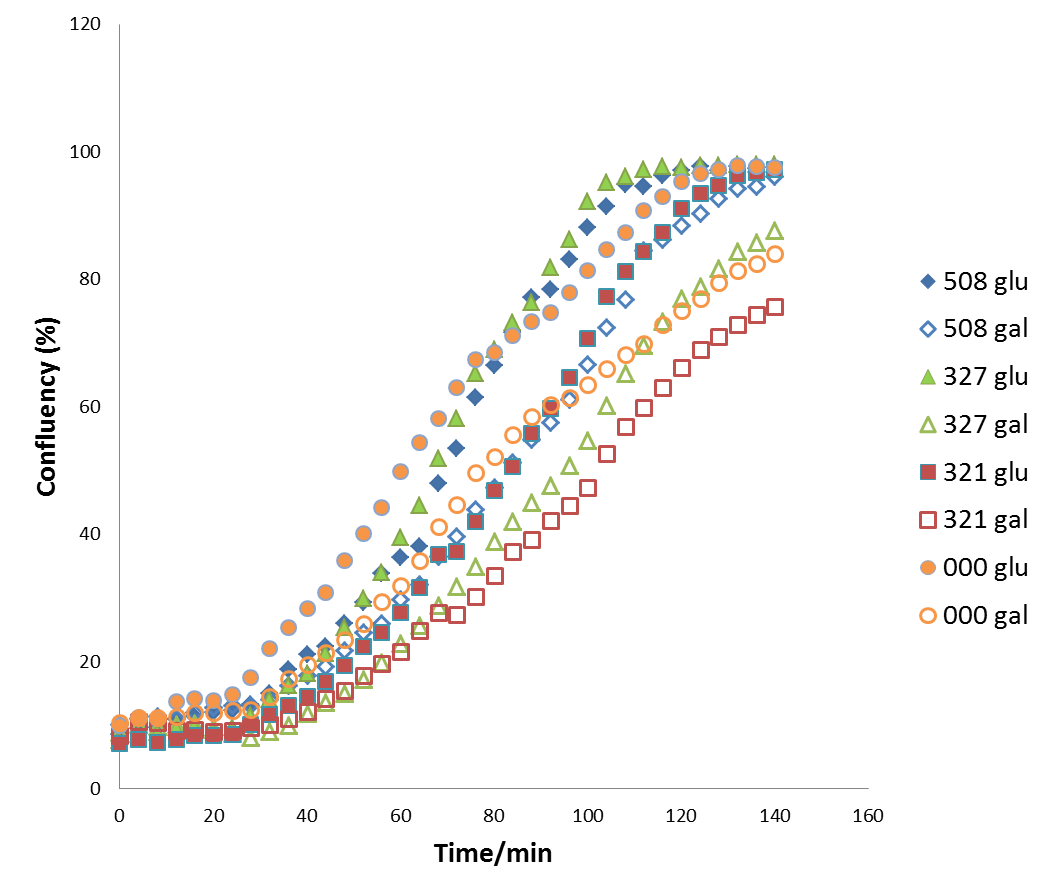


**S2 Figure. Reduced growth of patient fibroblasts in galactose medium**

Fibroblast lines from four different patients (A) and healthy volunteers (B) were cultured with either glucose (filled symbols) or galactose (open symbols) as a carbon source and confluency was measured in tripilcate with an Incucyte HD live cell imaging system. Patient derived cells needed on average 40 hours longer to reach 50% confluency in galactose compared to glucose. Control cells in galactose reached 50% confluency significantly faster, on average 19 hours later than glucose-grown cells (p=0.019).
