# Supplementary material for: Mutations in mitochondrial DNA causing tubulointerstitial kidney disease
Source: PLoS Genet. 2017 Mar 7;13(3):e1006620. doi: 10.1371/journal.pgen.1006620 (PMC5360345; doi:10.1371/journal.pgen.1006620)
Supplement: S3 Fig — Mitochondrial copy number was assessed by quantitative RT-PCR for the mitochondrial gene ND1 and the nuclear gene B2M and normalized to the mean ratio of healthy controls. As a group, patient-derived fibroblasts showed a significant increase in copy number relative to control fibroblasts (of different haplotypes). (DOCX) [file pgen.1006620.s003.docx]

S3 Figure


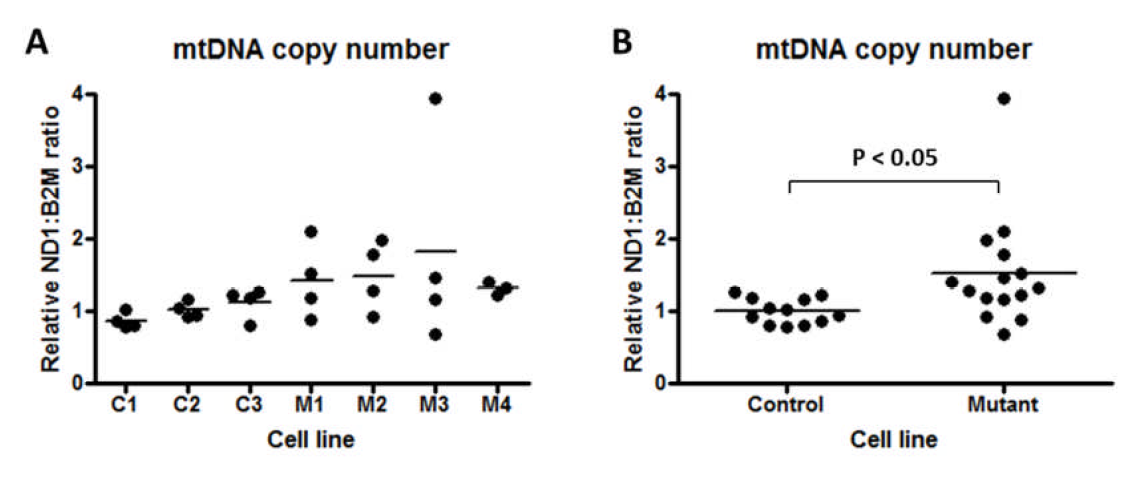


Figure S3: Increased mtDNA copy number in patient fibroblasts.

Mitochondrial copy number was assessed by quantitative RT-PCR for the mitochondrial gene ND1 and the nuclear gene B2M and normalized to a value of 1.0 for the mean ratio of healthy controls. As a group, patient-derived fibroblasts showed a significant increase in copy number relative to control fibroblasts (of different haplotypes).
