# Supplementary material for: Mutations in mitochondrial DNA causing tubulointerstitial kidney disease
Source: PLoS Genet. 2017 Mar 7;13(3):e1006620. doi: 10.1371/journal.pgen.1006620 (PMC5360345; doi:10.1371/journal.pgen.1006620)
Supplement: S4 Fig — Mitochondrial protein translation was analysed by blocking cytosolic translation with emetine in the presence of 35S methionine and cysteine. (A) A clear reduction of mitochondrial protein synthesis was observed in the patient-derived cybrids with the m.547A>T substitution. The characteristic bands of mitochondrial encoded proteins are annotated (ND1-6: NADH dehydrogenase subunit 1–6, CO I-III: mitochondrially encoded cytochrome c oxidase I-III, ATP6: mitochondrially encoded ATP synthase 6). (B) Total protein concentration of the radiolabelled samples was determined using TGX stain free gels (Bio Rad). (C)The scatter plot shows the mean, normalised mitochondrial protein production of patient and control cybrids with error bars indicating the standard deviation (p<0.01). (DOCX) [file pgen.1006620.s004.docx]

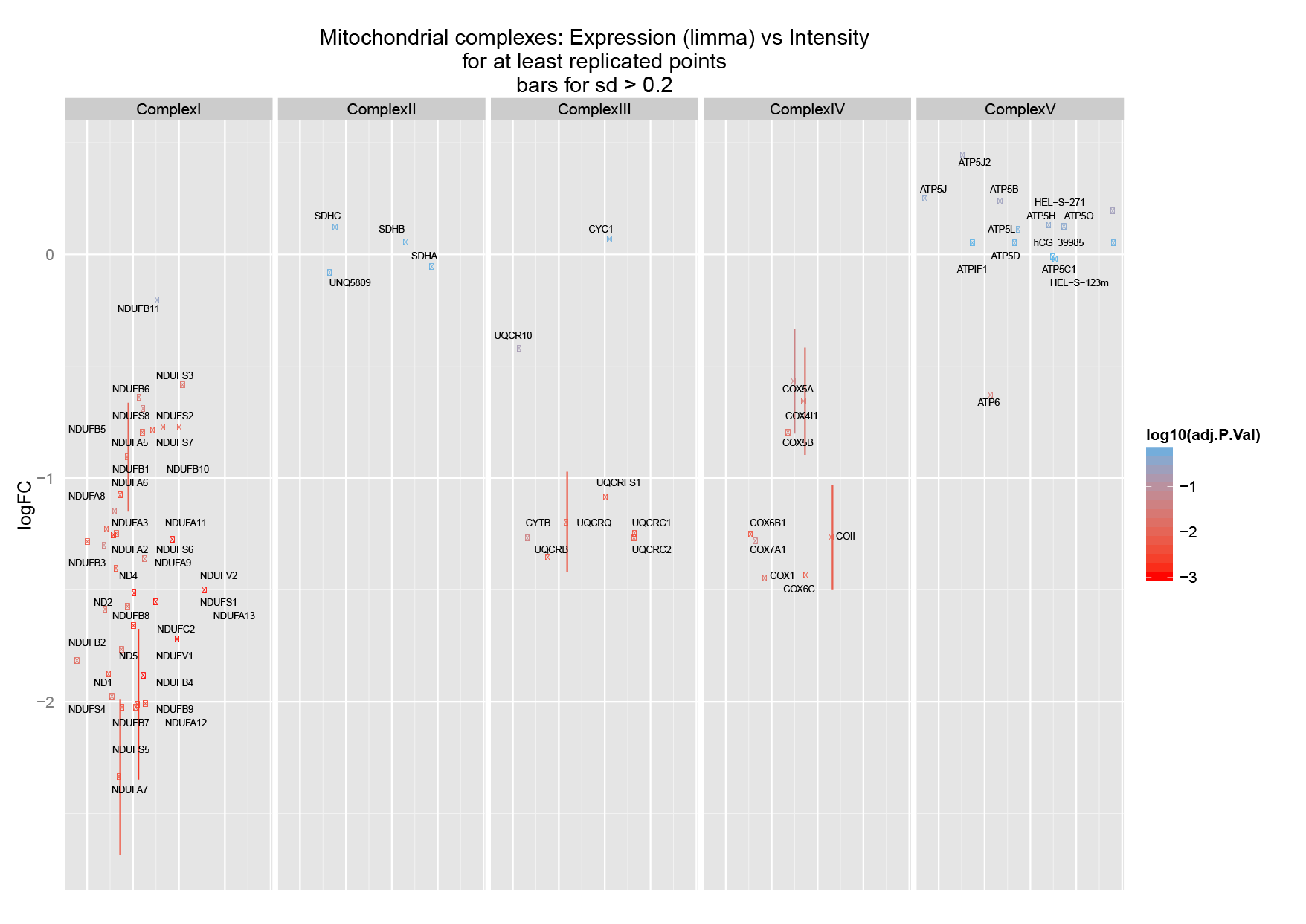


**S4 Figure: Fully annotated mitoSILAC data**

Individual subunits of the respiratory complexes identified in at least two of the four mitoSILAC experiments are listed. The binary logarithm of the fold change is shown (LogFC). Error bars are shown where the standard deviation exceeded 20% of the mean. The adjusted p value indicating significant changes is indicated by a blue to red colour scale. Uniprot gene names are given.
