# Supplementary material for: Mutations in mitochondrial DNA causing tubulointerstitial kidney disease
Source: PLoS Genet. 2017 Mar 7;13(3):e1006620. doi: 10.1371/journal.pgen.1006620 (PMC5360345; doi:10.1371/journal.pgen.1006620)
Supplement: S2 Table — Base indicates the position relative to the revised Cambridge reference sequence of human mitochondrial DNA; the GB frequency data is derived from 29,867 GenBank sequences with size greater than 15.4kbp and published on the MITOMAP database. A value of 1 indicates 100% prevalence [7]. (DOCX) [file pgen.1006620.s007.docx]

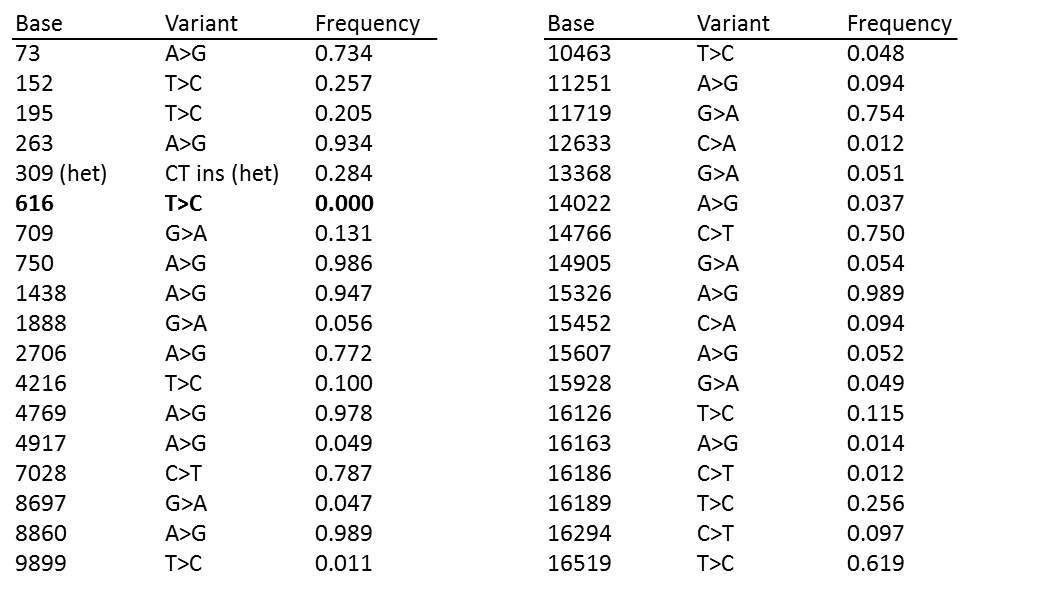


**S2 Table: mtDNA variants in pedigrees II and III**

Base indicates the position relative to the revised Cambridge reference sequence of human mitochondrial DNA; the GB frequency data is derived from 29,867 GenBank sequences with size greater than 15.4kbp and published on the MITOMAP database. A value of 1 indicates 100% prevalence.[**^7^**](#_ENREF_7).
