# Supplementary material for: Mutations in mitochondrial DNA causing tubulointerstitial kidney disease
Source: PLoS Genet. 2017 Mar 7;13(3):e1006620. doi: 10.1371/journal.pgen.1006620 (PMC5360345; doi:10.1371/journal.pgen.1006620)
Supplement: S3 Table — Biochemical analysis of skeletal muscle homogenate from a patient carrying the m.547A>T mutation showed that the activities of complexes I and IV are both outside the control range, while complexes II and III activities are normal. All enzyme activities are normalised for citrate synthase activity. Values outside the normal range are shown in bold. (DOCX) [file pgen.1006620.s008.docx]

**Table S3**


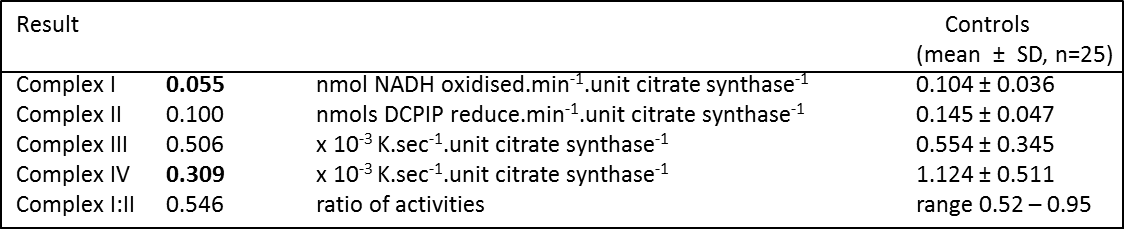


**Table S3: Muscle biopsy shows reduced complex I and IV activity**

Biochemical analysis of skeletal muscle homogenate from a patient carrying the m.547A>T mutation showed that the activities of complexes I and IV are both lower than controls, while complexes II and III activities are normal. All enzyme activities are normalised for citrate synthase activity. Values outside the control range are shown in bold
