# Supplementary material for: Mutations in mitochondrial DNA causing tubulointerstitial kidney disease
Source: PLoS Genet. 2017 Mar 7;13(3):e1006620. doi: 10.1371/journal.pgen.1006620 (PMC5360345; doi:10.1371/journal.pgen.1006620)
Supplement: S4 Table — Citrate synthase activity in total cell lysates was assessed by measuring the conversion of oxaloacetate and acetyl CoA to citrate and CoA-SH, which then reacts with dithio-nitrobenzoic acid (DTNB) to yield thio-nitrobenzoate which absorbs at 412 nm[41]. We analysed four different patient and control cell lines (3 technical repeats per experiment and the assay was repeated twice). The ratio of citrate synthase activity in patient and control fibroblast lines did not show a significant difference in any of the assays. (DOCX) [file pgen.1006620.s009.docx]

| relative CS activity | Assay 1 | Assay 2 | Assay 3 |
| --- | --- | --- | --- |
| patient/control | 1.069640239 | 0.8872667 | 1.11468039 |
| t-test | 0.114914326 | 0.0825953 | 0.19639883 |

S4 table: Citrate synthase activity of patient and control fibroblasts

Citrate synthase activity in total cell lysates was assessed by measuring the conversion of oxaloacetate and acetyl CoA to citrate and CoA-SH, which then reacts with dithio-nitrobenzoic acid (DTNB) to yield thio-nitrobenzoate which absorbs at 412 nm (Kirby et al 2007). We analysed four different patient and control cell lines (3 technical repeats per experiment and the assay was repeated twice). The ratio of citrate synthase activity in patient and control fibroblast lines did not show a significant difference in any of the assays.
